# Case Report: History‐Indicated McDonald’s Cerclage for Uterine Didelphys and History of Cervical Insufficiency

**DOI:** 10.1155/crog/9951473

**Published:** 2025-12-11

**Authors:** Eleanore Rominger, Henry Tal Lesser, Fereshteh Boozarjomehri

**Affiliations:** ^1^ Geisinger Health System, Department of Obstetrics and Gynecology, Danville, Pennsylvania, USA, geisinger.org

## Abstract

**Introduction:**

Mullerian anomalies alone are not an indication for prophylactic and history indicated cerclage (HIC). Data on the use of cerclage for patients with mullerian anomalies is therefore limited to case reports of ultrasound indicated cerclage (UIC) and physical exam indicated cerclage (PIC). Given these limitations, there is a hesitancy towards cerclage use in this subset of patients. This case report aims to highlight that HIC is a safe and effective intervention for patients with mullerian anomalies and a history of cervical insufficiency (CI).

**Case:**

We present a case of a patient with uterine didelphys and a history of CI that underwent a successful McDonald HIC in a subsequent pregnancy. Surveillance throughout pregnancy was reassuring and the patient delivered preterm at 32 weeks and 0 days’ gestation with a favorable final outcome.

**Conclusion:**

Although data is limited regarding cerclage use in patients with mullerian anomalies, this case suggests that a HIC is a low risk and effective intervention that should not be withheld from this subgroup of patients.

## 1. Introduction

Cervical insufficiency (CI) is a rare and potentially preventable pregnancy related complication that contributes significantly to premature births and related adverse perinatal outcomes. History indicated cerclage (HIC) or cervical length screening with ultrasound indicated cerclage (UIC) are offered to patients with a history of preterm birth based specific circumstances [1]. Despite an increased risk for preterm birth from both preterm labor and/or CI, patients with mullerian anomalies, including uterine didelphys (UD), are often not included in this treatment algorithm because of lack of evidence supporting its use. In this case report, we present a case that strengthens the claim that patients with UD are at risk for CI and therefore, a HIC should be offered in this subset of patients, as it is low risk and may increase the likelihood of a favorable pregnancy outcome.

## 2. Case

This is a case of a 21‐year‐old G2‐P0100, with UD and a history of CI, who underwent HIC. The patient was diagnosed with UD during her first pregnancy. That pregnancy was in the right uterine horn, and a routine mid‐second trimester ultrasound at 19 weeks and 4 days was normal aside from an incidental immeasurable cervix with concern for dilation. Speculum examination confirmed a 3‐cm dilated cervix and a physical exam indicated cerclage (PIC) was not offered. The patient opted for expectant management but presented later that day in preterm labor and experienced an otherwise uneventful second trimester loss.

Six months later, the patient became pregnant and was referred to maternal–fetal medicine (MFM). Ultrasound demonstrated a normal intrauterine pregnancy (contralateral horn, left) and a transvaginal cervical length of 37.3 mm (see Figure 1). Through shared decision‐making, a HIC was planned at 15 weeks. Careful review of the literature was undertaken, which confirmed a paucity of data on cerclage placement in patients with mullerian anomalies, but specifically only one case of HIC for UD [2, 3]. The planned surgical approach was a McDonald’s purse string technique using a Mersilene polyester fiber strip 5 mm in diameter. Intraoperatively, ultrasound was used to confirm the appropriate cervix connecting to the pregnant uterus was being sutured. Physical exam revealed a longitudinal vaginal septum separating two cervices. The left cervix for the uterine horn containing the pregnancy was grasped. The cervical‐vesico reflection was visualized and in standard fashion a McDonald’s cerclage was placed high on the cervix (see Figure 2) [1].

Figure 1Pre‐cerclage placement imaging demonstrating uterine didelphys (a and b). Transvaginal ultrasound of the left cervical length measuring 37.7 mm for the uterine horn containing the pregnancy (c).

**Figure:**
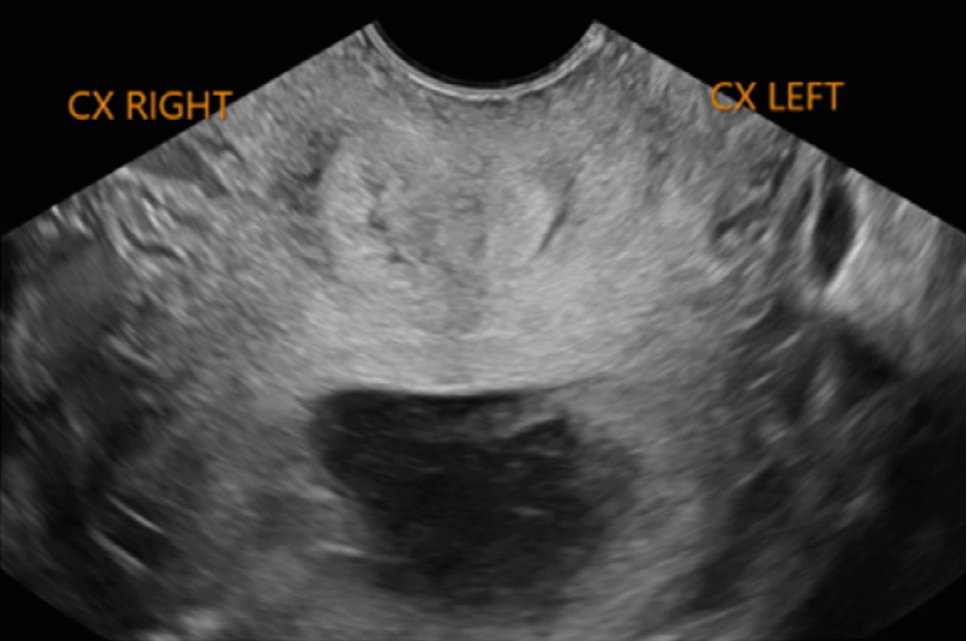
(a)

**Figure:**
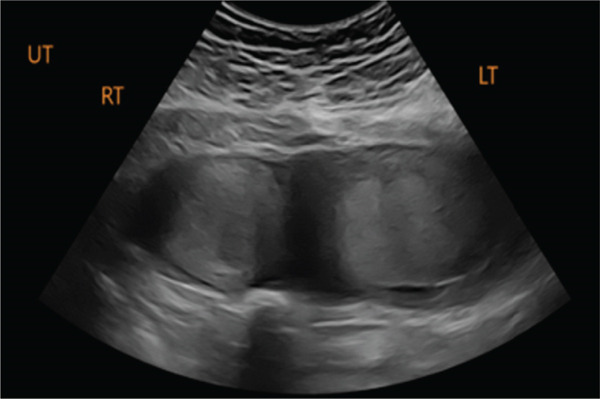
(b)

**Figure:**
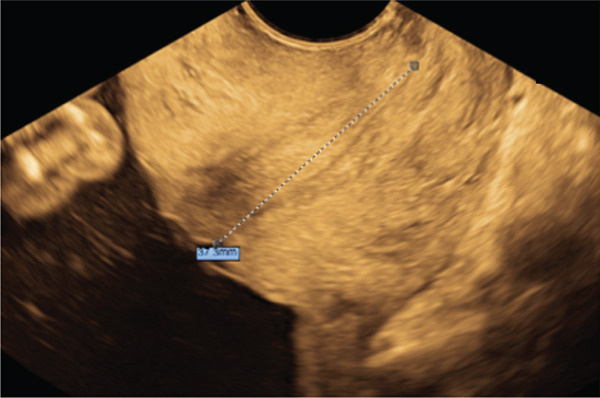
(c)

Figure 2Intraoperative views demonstrating longitudinal vaginal septum separating right and left cervices (a). Post‐cerclage image of the left cervix (b).

**Figure:**
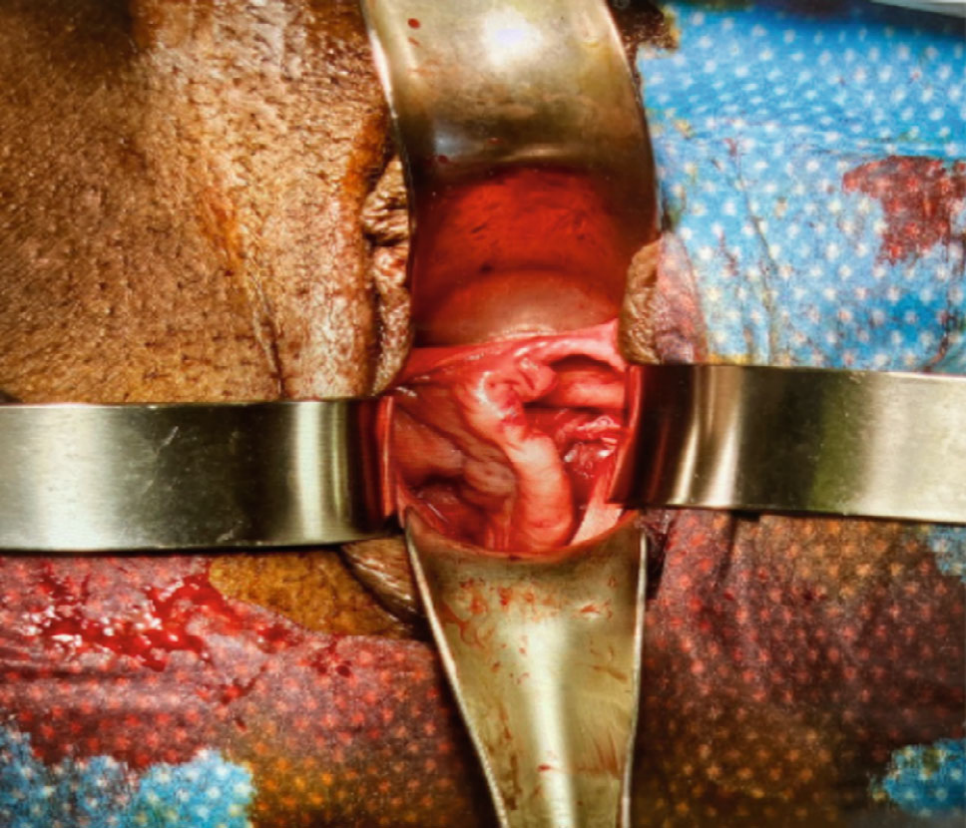
(a)

**Figure:**
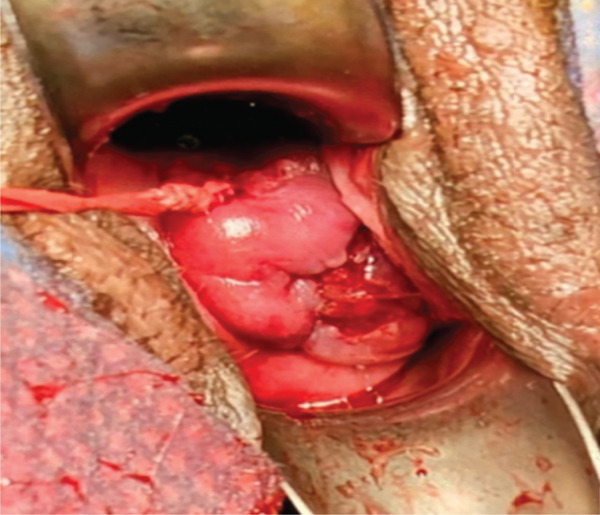
(b)

**Figure 3:**
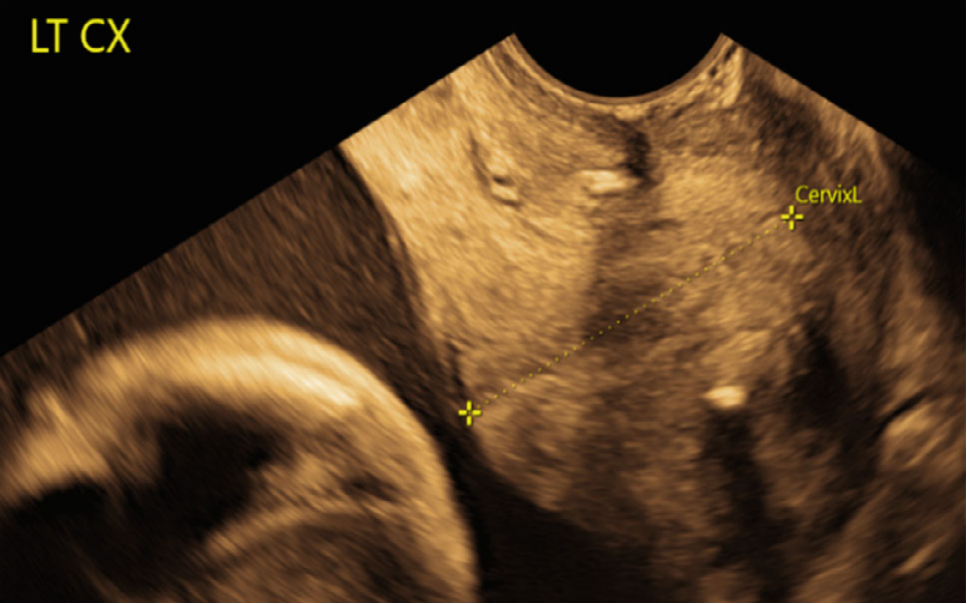
Two weeks post‐cerclage placement follow‐up transvaginal ultrasound demonstrating 35.5 mm functional residual cervix and intact cerclage placement (left cervix).

Repeat imaging after 2 weeks revealed an intact cerclage with a residual cervical length measuring 35.5 mm (see Figure 3). Serial growth scans during pregnancy were unremarkable. The patient presented to labor and delivery with contractions at 27 weeks and 5 days gestation. No cervical trauma or evidence for preterm labor was noted; however, betamethasone was administered with an abundance of caution given the patients prior history. Tocolysis was not administered, and the patient was subsequently discharged with a diagnosis of Braxton–Hicks contractions. Four weeks later, the patient returned in preterm labor. The cerclage was in place and no cervical trauma was seen. The suture was removed atraumatically and without difficulty. An uncomplicated preterm delivery followed at 32 weeks 0 day (108 days post‐cerclage placement). A vigorous male infant was born weighing 2520 g with APGARS of 2, 5, and 7 and an estimated blood loss of 450 mL. The infant remained in the neonatal intensive care unit for 19 days before being discharged with no apparent complications. The patient was seen at a postpartum visit where she endorsed meeting all milestones, reported a healthy child, and was exceptionally thankful and appreciative for the care she received and the outcome of this pregnancy.

## 3. Discussion

Mullerian anomalies complicate approximately 0.5% of all pregnancies and contribute greatly to adverse perinatal outcomes, most notably early pregnancy loss and preterm birth [4]. While this is true for all mullerian anomalies, although rare, UD accounts for a disproportionately higher rate of these outcomes [4].

Distinguishing between preterm labor and painless cervical dilation resulting in labor can be challenging. In subsequent pregnancies, those who previously experienced preterm labor are offered serial cervical length screening with UIC as needed, while patients with CI are recommended to have a HIC placed earlier [1]. When comparing cervical length surveillance versus history‐based intervention, surveillance resulted in a greater number of cerclages and intervention with no improvement in outcomes. This study concluded that ultrasound surveillance does not improve selection for cerclage and a short cervix alone is not a reliable indicator for cerclage in all at‐risk patients [5]. When used, HIC can have significant advantages over UIC including fewer perioperative complications as well as better perinatal outcomes including a decreased rate of spontaneous preterm birth and these findings were also confirmed in prospective randomized trials [6, 7]. Data regarding any cerclage, but especially HIC, is lacking in patients with mullerian anomalies. Most data focus on UIC and patients with bicornuate uterus. For these patients, compared with controls, cerclage was associated with a significant reduction in preterm birth [3]. Although data regarding its use in patients with mullerian anomalies, especially UD, is limited, we suggest that a HIC should therefore not be withheld from such patients given its significant potential benefit with no known additional risk [2, 3]. Surgical approaches vary and there is heterogeneity in the study population regarding indication and technique. Preoperatively, we reviewed the literature specifically for cerclage and UD, and cases of successful laparoscopic and open transabdominal HIC, as well as an UIC using McDonald’s technique, were identified [2, 3]. Since transabdominal cerclage has unique considerations as well as increased pregnancy related morbidity, this team elected to proceed with a McDonald’s technique [1]. Despite anatomical variations, we were able to place a cervical cerclage with little difficulty and a follow‐up ultrasound confirmed optimal cerclage location with over 3 cm of functional residual cervix remaining.

In this case of a patient with UD and a history of CI, the use of HIC resulted in a preterm delivery at nearly 33 weeks with no additional complications. To our knowledge, this is the first case describing a HIC using McDonald technique in a patient with UD. The cerclage was placed by the same team that managed her pregnancy and was present for delivery and so no details of the clinical course are absent. We also recognize that there are limitations to this report including that the two pregnancies (2nd trimester loss, and subsequent pregnancy with cerclage) were in two different uterine horns and so it is possible that the risk for CI or preterm birth differed by pregnancy location. Additionally, some may consider preterm birth at 32 weeks an adverse outcome with HIC. Cerclages, especially HIC, are a low‐risk and high‐reward intervention to prevent pregnancy loss in patients with CI [6]. As seen in this case, for patients with mullerian anomalies, including UD, providers should not be discouraged from offering any cerclage, including HIC.

## Patient Perspective

“Losing my first pregnancy after finding out my cervix had opened without any warning was heartbreaking. I was scared to get pregnant again, and the uncertainty and lack of clear consensus guidelines for managing a condition like mine was concerning. The different options were laid out for me clearly and through shared decision making my team and I agree to pursue a cerclage. I always felt like my team listened to my concerns and cared about me and my baby. Even when I started having contractions early, they were calm and reassuring. Delivering at 32 weeks was hard, but my baby did great and is healthy. I’m so thankful for the amazing care and guidance I received from my team throughout the whole experience.”

## Conflicts of Interest

The authors declare no conflicts of interest.

## Funding

No funding was received for this manuscript.

## Supporting information


**Supporting Information CARE** Additional supporting information can be found online in the Supporting Information section. Guidelines Checklist.

## Data Availability

As this is a case report, there is no data interpreted to support the findings of this clinical case. Patient consent was obtained prior to the writing or submission of this case report.
