# Supplementary material for: Case Report: History‐Indicated McDonald’s Cerclage for Uterine Didelphys and History of Cervical Insufficiency
Source: Case Rep Obstet Gynecol. 2025 Dec 11;2025:9951473. doi: 10.1155/crog/9951473 (PMC12747113; doi:10.1155/crog/9951473)
Supplement: Supplementary file 1 — Supporting Information CARE Additional supporting information can be found online in the Supporting Information section. Guidelines Checklist. [file CROG-2025-9951473-s001.pdf]

CARE case report guidelines

CARE Checklist of information to include when writing a case report

(cc) BY-NC-ND

Topic	Item	Checklist item description	Reported on Line
Title	1	The diagnosis or intervention of primary focus followed by the words "case report"	1-2
Key Words	2	2 to 5 key words that identify diagnoses or interventions in this case report, including "case report"	29-31
Abstract (no references)	3a	Introduction: What is unique about this case and what does it add to the scientific literature?	29-31
	3b	Main symptoms and/or important clinical findings	33-36
	3c	The main diagnoses, therapeutic interventions, and outcomes	33-36
	3d	Conclusion—What is the main "take-away" lesson(s) from this case?	38-40
Introduction	4	One or two paragraphs summarizing why this case is unique (**may include references**)	122-128
Patient Information	5a	De-identified patient specific information.	59-60
	5b	Primary concerns and symptoms of the patient.	59-69
	5c	Medical, family, and psycho-social history including relevant genetic information	59-69
	5d	Relevant past interventions with outcomes	64-65
Clinical Findings	6	Describe significant physical examination (PE) and important clinical findings.	67-68
Timeline	7	Historical and current information from this episode of care organized as a timeline	155-156
Diagnostic Assessment	8a	Diagnostic testing (such as PE, laboratory testing, imaging, surveys).	67-68
	8b	Diagnostic challenges (such as access to testing, financial, or cultural)	69-72
	8c	Diagnosis (including other diagnoses considered)	66-68
	8d	Prognosis (such as staging in oncology) where applicable	NA
Therapeutic Intervention	9a	Types of therapeutic intervention (such as pharmacologic, surgical, preventive, self-care)	72-73
	9b	Administration of therapeutic intervention (such as dosage, strength, duration)	72-78
	9c	Changes in therapeutic intervention (with rationale)	68-72
Follow-up and Outcomes	10a	Clinician and patient-assessed outcomes (if available)	85-92
	10b	Important follow-up diagnostic and other test results	89-92
	10c	Intervention adherence and tolerability (How was this assessed?)	79-86
	10d	Adverse and unanticipated events	81-84
Discussion	11a	A scientific discussion of the strengths AND limitations associated with this case report	
	11b	Discussion of the relevant medical literature **with references**.	98-121
	11c	The scientific rationale for any conclusions (including assessment of possible causes)	122-127
	11d	The primary "take-away" lessons of this case report (without references) in a one paragraph conclusion	122-127
Patient Perspective	12	The patient should share their perspective in one to two paragraphs on the treatment(s) they received	147-154
Informed Consent	13	Did the patient give informed consent? Please provide if requested	Yes ☒ No ☐
